# Effects of Alcoholism upon the Nervous System

**Published:** 1873-06

**Authors:** 


### ARTICLE VII.

### *Effects of Alcoholism upon the Nervous System.*

A few months since we called the attention of our readers to the demonstrated facts concerning the physiological action of alcohol. We wish now to give some of the effects that its continuous use produces in the nervous system. It must be remembered that this system is composed essentially of nerve cells, nerve fibres, blood vessels, connective tissue and unceasing membranes. The nerve cells and nerve fibres perform the work of the nervous system, the other elements merely protect and nourish these. Now what effect has the use of alcohol upon these anatomical elements ?

In answering this question we cannot do better than to give the statements of Dr. Austin, as published in the *Lancet* for February, 1873 :

In the brain of old drinkers there is found a general shrinking of the cerebral mass, a flattening of the convolutions, and the arachnoid space. Microscopic sections show that the same atrophy extends to the individual nervous elements of the centers, while the connective tissue is everywhere thickened. Besides the development of fibroid connective tissue there is much granular fat, representing the the destruction of the nervous elements. True inflamations are seen in dura mater in the neighborhood of the longitudinal sinus, and of the pia mater at various points along the line of the vessels. In cases of drink of any standing, the smaller arteries and capillaries are markedly affected with atheroma. In many cases the sheathing membrane is distended with the remnants of effused blood, and its external surface surrounded with heaps of minute oil globules. Moreover at particular points in the course of a vessel, the surrounding nervous tissues are dotted with punctform hemorrhages. To sum up: The unvarying effect of continuous impregnation of the blood supplied to the brain, with considerable per centages of alcohol, are degenerated and atrophy of the vessels, thickening of the connective tissue elements, and in the smaller number of cases inflammation of the membranes, which in the case of dura mater tends to be limited in extent, but in that of the pia mater may be diffused over a considerable surface; but in all cases there is a conspicuous atrophy of the true nervous elements, more especially of the vesicular matter.

In short, alcohol destroys the nerve cells and fibres, disables or destroys the vessels which supply the blood to nourish the parts, and cause an increase of connective tissue. No wonder that there is a perversion of the mental function when the organs through which they alone can work are thus crippled.—*Detroit Review of Medicine.*

